# Why are hospitalisations too long? A simple checklist for identifying the main social barriers to hospital discharge from a nephrology ward

**DOI:** 10.1186/s12882-018-1023-1

**Published:** 2018-09-12

**Authors:** Jean Philippe Coindre, Romain Crochette, Conrad Breuer, Giorgina Barbara Piccoli

**Affiliations:** 1 Néphrologie, Centre Hospitalier Le Mans, 194 Avenue Rubillard, 72000 le Mans, France; 2 Direction des Finances, du Système d’Information et du Contrôle de Gestion, Centre Hospitalier Le Mans, 72000 le Mans, France; 3 Dipartimento di Scienze Cliniche e Biologiche, Università di Torino, Turin, Italy

**Keywords:** Hospitalisation, Social check-list, Cost benefit, Economic analysis, Social barriers, Elderly patients, Efficacy, Efficiency, Healthcare system

## Abstract

The present increase in life span has been accompanied by an even higher increase in the burden of comorbidity. The challenges to healthcare systems are enormous and performance measures have been introduced to make the provision of healthcare more cost-efficient. Performance of hospitalisation is basically defined by the relationship between hospital stay, use of hospital resources, and main diagnosis/diagnoses and complication(s), adjusted for case mix. These factors, combined in different indexes, are compared with the performance of similar hospitals in the same and other countries. The reasons why an approach like this is being employed are clear.

Cutting costs cannot be the only criteria, in particular in elderly, high-comorbidity patients: in this population, although social issues are important determinants of hospital stay, they are rarely taken into account or quantified in evaluations. Quantifying the impact of the “social barriers” to care can serve as a marker of the overall quality of treatment a network provides, and point to specific out-of-hospital needs, necessary to improve in-hospital performance. We therefore propose a simple, empiric medico-social checklist that can be used in nephrology wards to assess the presence of social barriers to hospital discharge and quantify their weight.

Using the checklist should allow: identifying patients with social frailty that could complicate hospitalisation and/or discharge; evaluating the social needs of patient and entourage at the beginning of hospitalisation, adopting timely procedures, within the partnership with out-of-hospital teams; facilitating prioritization of interventions by social workers.

The following ten items were empirically identified: reason for hospitalisation; hospitalisation in relation to the caregiver’s problems; recurrent unplanned hospitalisations or early re-hospitalisation; social/family isolation; presence of a dependent relative in the patient’s household; lack of housing or unsuitable housing/accommodation; loss of autonomy; lack of economic resources; lack of a safe environment; evidence of physical or psychological abuse.

The simple tool here described needs validation; the present proposal is aimed at raising attention on the importance of non-medical issues in medical organisation in our specialty, and is open to discussion, to allow its refinement.

## Background

The world population is ageing and life expectancy is presently over 80 in many countries. However, this is not without a price: the increase in longevity has been accompanied by an even higher increase in comorbidity [1, 2].

The present crisis, affecting all European countries’ economies, is posing further challenges to their healthcare systems, called upon to respond to increased demand for care with reduced budgets, a trend that is not likely to be reversed in the near future [3].

While performance criteria are needed to ensure equity and efficiency in healthcare, no performance measure is fully satisfactory [4]. Each measurement has limits and none is able to consider the system as a whole. Dealing with performance control can be difficult for clinicians, many of whom feel it involves a conflict with their role as patients’ advocates. In addition, clinicians generally do not have management training and often consider that time devoted to this activity could be better spent in clinical practice [4–7].

Understanding the mechanisms and analysing the critical points may however allow us to prevent dangerous budget cuts, redefining how control of the process of delivery of care should be approached.

Performance of hospitalisation is basically defined by the relationship between hospital stay and/or use of hospital resources, and main diagnosis/diagnoses and complication(s), adjusted for case mix (mainly age and comorbidity). These factors, combined in different indexes, are compared to the average performance of similar hospitals or clinics in the same and other countries [8–14].

The mantra of the new millennium is that “healthcare needs to go home”: the majority of patients can be treated in ambulatory services and hospital stays should be only for the most demanding cases [15, 16]. In Nephrology, at different speeds, with different degrees of efficiency and at different levels, most European countries have supported the development of outpatient facilities and home-based treatments. However, a sensible increase in need for hospitalisation is foreseen in Nephrology in at least some European Countries (+ 4.8% in France for patients aged over 75 years, in the 2017 document addressed at projections for 2030) [17].

Where home based-treatments, outpatient units and day-hospital facilities are well developed, the selection of the patients who are hospitalised is stricter and, even in the presence of similar diagnoses and comorbidity, only the most severe cases and those with the most complex support needs (including social problems) are actually hospitalised [18, 19]. In this regard, we may identify a curious paradox, since the “worst” hospitalisation results (in which duration of the hospitalisation is the main criterion of evaluation) may be recorded in the “best” systems, and within each system, the centres with the best out-of-hospital networks may be penalised by their own efficiency. A simple solution for correcting the prevalence of out-of-hospital activity is probably not satisfactory in a field in which up to 40% of the patients start dialysis in emergency and without regular nephrology follow-up [20–28].

Furthermore, the clinical and social issues are not separate in the evaluation process, and clinicians are obviously more efficient in addressing clinical problems; as a consequence, good clinical activity may be penalised by lack of social resources, a situation which clinicians have few way of affecting. The relative importance of the availability of home assistance and structures providing out-of-hospital care is not jet quantified.

Starting from this premise, we have prepared a simple, quick medico-social checklist to assess the presence of social barriers to hospital discharge from a nephrology ward and quantify their weight.

### The role of “social” problems in hospital stay

An interim analysis of the performance indexes recorded in our nephrology unit, reported in Table 1, exemplifies a common situation in France: most patients hospitalised for long periods are old, have high comorbidity, and are affected by multiple failures/problems, as witnessed by the fact that 25% of the cases have been hospitalised in more than one ward, including intensive care.

**Table 1:** Main critical features in the discharge of 20 patients hospitalised for at least 35 days in a nephrology ward

Comorbidity- risk factor	Prevalence
Age > =75 years	90% (18/20)
Hospitalisation in multiple hospital wards	75% (15/20)
At least one severe comorbidity	75% (15/20)
Charlson Index	8 (6–13)
Complications during hospitalisation	70% (14/20)
Need for dialysis or dialysis dependence	40% (8/20)
Social factor/s delaying hospital discharge for > 1 week	25% (5/20)

Note: analysis of the 20 longest hospital stays observed in 2017 in a 16-bed nephrology unit in the 1750-bed hospital, in Le Mans, France. The hospital serves a catchment area with about 300,000 inhabitants and its nephrology beds are the only ones available in an area with approximately 700,000 inhabitants. The centre has daily outpatient consultations (about 3000 in 2017) and an active day hospital in which 350 patients were treated in 2017

The principal social problems which delayed discharge: difficulties involved in return to family (patient “too heavy”, dependent, bedridden..); lack of institutional solution (no availability of downstream beds); problems connected to dialysis (transport costs, clinical complexity); insufficient cooperation with geriatricians (overworked teams)

When we tried to analyse the weight of social problems in hospital stay, we realised that only the most relevant ones and only the longest delays are recorded, for example those due to a lack of out-of-hospital structures. As a consequence, we were not fully able to assess in retrospect the weight of “minor”, albeit possibly systematic delays in hospital discharge due to social difficulties or organizational factors. This is not irrelevant if we take into account that one-day of delay per hospitalisation can profoundly influence the performance indexes of a nephrology ward.

While, from the clinical point of view we decided to refine the patient description systematically using Charlson’s Comorbidity Index and the Karnofsky performance Status Scale, we were not able to find a similar tool to rapidly and systematically assess the social barriers to hospital discharge, to quantify their weight in the overall hospitalisation performance [29, 30].

### A simple medico-social checklist for identifying “social” barriers to hospital discharge

A checklist is a useful potential tool to identify problems or proposing solutions; it should be concise, rapid and pragmatic, as it is aimed at securing crucial procedures, such as in flying airplanes, more than at assessing details [31–33]. We therefore tried to identify a limited number of items to assess when a patient is admitted or transferred into a nephrology ward, to facilitate:

- early identification of patients with social frailty that could complicate hospitalisation and delay discharge;
- timely evaluation of the social needs of the patient and his/her entourage, in order to adopt a multidisciplinary approach that includes partnership with out-of-hospital teams;
- prioritization of interventions by social workers (evaluation of the level of risk).

Ten items were empirically selected by the members of the nephrology team, after extensive discussion (Table 2). They are by no means exhaustive, but we feel they include the situations most often encountered in our setting, while the option “notes” was provided to record unforeseen situations, to enable us to refine the list over time. Some of the items are at least partially overlapping (i.e. difficult home maintenance is usually a result of loss of autonomy and may be accompanied by social isolation, or be the result of clinical fragility, which is also reflected in recurrent hospitalisation); this was chosen to enable a cross control of the most relevant ones and to enhance their relative weight.

**Table 2:** A medico-social checklist for identifying the main social barriers in hospital discharge from a nephrology unit

Item	If item is selected = need to evaluate social care	select
1	Reason for hospitalisation = difficult (or impossible) to care for patient at home.	
2	Hospitalisation in relation to the caregiver’s problems (hospitalisation, caregiver burnout, other).	
3	Recurrent unplanned hospitalisations (> 1 per month), early readmissions (< 8 days)*	
4	Social/family isolation (living alone or lack of support from entourage).	
5	Presence of a dependent relative or minor left alone as a result of the patient’s hospitalisation	
6	Lack of housing/accommodation or accommodation temporarily or permanently unsuitable (unhealthy, inadequate...).	
7	Loss of autonomy in the acts of daily life.	
8	Unemployed or lack of economic resources.	
9	Patient does not receive social security benefits, therefore unable to pay for medical expenses (drugs, nursing, physiotherapy, etc).	
10	Patient known or suspected to be subject to abuse.	
Action	Please record and complete to allow quantification	
1	Date of social worker’s call ………. Date of report……..	
2	Date of acceptance in an out-of-hospital care facility.	
3	Days from clinical indication for hospital discharge to actual hospital discharge	

*Intervals based upon the usual analysis in our setting [40]

In fact, in its present form, the checklist is intended to identify cases in which social support is needed, and not to grade the degree of severity of the social problem, whose solutions mainly depend on an out-of-hospital network of care.

Conversely, since all the items recorded were associated with patient survival in the overall population and in different fields of internal medicine, we felt that this dataset could be of interest for the long-term evaluation of the “social” risks and “social disease” burden in our population, complementing the clinical comorbidity indexes mentioned above [34–38].

### Perspectives: the traditional role of the physician is changing

We are no longer “just” doctors, but are being called upon to be managers, experts in communications and opinion leaders. Expertise in hospital management, statistics and law is increasingly required, and physicians are increasingly being asked to assume non-medical responsibilities [6, 7]. The optimisation of the duration of hospital stay is one example.

From the clinical point of view, in an ideal setting of unlimited hospital resources, if a patient were to remain in the hospital one or two days longer than necessary (for example, so that home care could be organised), this would not be relevant, while in the presence of limited resources small systematic differences can finally result in suboptimal care for patients who “compete” for hospital services.

Even when physicians assume the responsibility for the optimisation of resources, making it possible to treat more patients with the same resources, their influence on non-medical factors is limited, since management is either in other hands (e.g. social workers’) or involves competition to obtain a greater share of the limited resources available (e.g. competition with other wards for placing patients in nursing homes).

Control measures are often “punitive” and risk holding the physician responsible for the functioning of the entire network; since resources are often allocated in relation to efficiency, the settings treating the most complex cases, with more need for out-of-hospital care, may be the ones that are penalised. In such a setting, quantifying the impact of the “social barriers” to care can serve as a marker of the overall quality of treatment a network provides, and point to specific out-of-hospital needs, necessary to improve in-hospital performance.

Furthermore, since virtually all the markers on our checklist are acknowledged survival markers, their quantification can contribute to defining “social comorbidity”, a missing determinant in the characterization of critical populations such as the ones that are routinely followed in our nephrology wards [39].

## Conclusions

One of the challenges healthcare systems are currently facing is reaching a reasonable compromise between delivering care to the maximum number of individuals and promoting excellence, which is the basis of progress and clinical research. Attaining a balance between full satisfaction of patients’ needs and reasonable costs is not easy, and the weight of non-medical factors (social barriers, economic constraints, logistic needs) can be at least as important as medical factors in attaining compliance, efficacy and treatment satisfaction.

In the case of hospitalisation, a detailed analysis of the social barriers to hospital discharge can help us assign the correct responsibilities to each healthcare intervenient (physician, social worker, healthcare manager), and thereby improve care despite cost constraints. The simple tool described in this opinion paper needs validation; the present proposal is aimed at raising attention, and is open to discussion, to allow its refinement.
